# Voluntary stopping of eating and drinking: is medical support ethically justified?

**DOI:** 10.1186/s12916-017-0950-1

**Published:** 2017-10-20

**Authors:** Ralf J. Jox, Isra Black, Gian Domenico Borasio, Johanna Anneser

**Affiliations:** 1 Institute for Ethics, History and Theory of Medicine, University of Munich, Lessingstr. 2, D-80336 Munich, Germany; 2 Geriatric Palliative Care, University of Lausanne, Lausanne, Switzerland; 3 York Law School, The University of York, York, UK; 4 Palliative Care Service, Technical University Munich, Munich, Germany; 5 Service de Soins Palliatifs et de Support, Centre Hospitalier Universitaire Vaudois, Lausanne, Switzerland

**Keywords:** Decision making, Nutrition and hydration, Palliative care, Ethical analysis, Medical ethics, Legal aspects

## Abstract

**Background:**

Physician-assisted dying has been the subject of extensive discussion and legislative activity both in Europe and North America. In this context, dying by voluntary stopping of eating and drinking (VSED) is often proposed, and practiced, as an alternative method of self-determined dying, with medical support for VSED being regarded as ethically and legally justified.

**Argument:**

In our opinion, this view is flawed. First, we argue that VSED falls within the concept of suicide, albeit with certain unique features (non-invasiveness, initial reversibility, resemblance to the natural dying process). Second, we demonstrate, on the basis of paradigmatic clinical cases, that medically supported VSED is, at least in some instances, tantamount to assisted suicide. This is especially the case if a patient’s choice of VSED depends on the physician’s assurance to provide medical support.

**Conclusion:**

Thus, for many jurisdictions worldwide, medically supported VSED may fall within the legal prohibitions on suicide assistance. Physicians, lawmakers, and societies should discuss specific ways of regulating medical support for VSED in order to provide clear guidance for both patients and healthcare professionals.

Please see related article: http://bmcmedicine.biomedcentral.com/articles/10.1186/s12916-017-0951-0.

## Background

Severely ill patients with a short life expectancy may have the desire to hasten death [1, 2]. Yet, euthanasia and assisted suicide (together called ‘assisted dying’) are legally prohibited in most countries, despite a recent liberalization in some North American jurisdictions [3, 4]. Therefore, patients may resort to another option to hasten their death – voluntary stopping of eating and drinking (VSED) [5] – wherein patients deliberately and voluntarily cease eating and/or drinking to bring about their own death earlier than it would have occurred naturally [6]. Studies show that the prevalence of VSED is underestimated and may in fact be higher than that of assisted suicide [7, 8]. Although the dying process is generally reported to be peaceful, symptoms such as thirst, pain, insomnia, anxiety, and delirium may require medical support [7–9]. Professional medical organizations, while rejecting assisted dying, are increasingly advocating VSED (and medical support for VSED) without giving a convincing ethical justification [10–12]. Most Western jurisdictions seem to permit medical support for VSED [13], even in jurisdictions where assisted dying is prohibited by law, such as England and (partially) Germany [14, 15]. However, a clear legal basis for medically supported VSED in statute or common law is often lacking. Indeed, healthcare professionals often express moral uncertainty as to whether medical support in the context of VSED constitutes suicide assistance [16].

In this article, we first show that VSED should be categorized, on a purely descriptive basis, as a form of suicide, albeit with particular characteristics that are not shared by other forms. Second, we argue that supporting patients who embark on VSED amounts to assistance in suicide, at least in some instances, depending on the kind of support and its relation to the patient’s intention. Third, we conclude that, given the ethical equivalence of supported VSED and assisted suicide in some cases, consistency is required in either allowing or restricting both forms of aid in dying, depending on the normative grounds for justification. This will have significant repercussions for the ethics codes of the medical profession as well as the law in several jurisdictions.

## Argument

### VSED and suicide

In colloquial language, suicide is understood as the act of intentionally taking one’s own life [17]. Legal definitions usually focus on the action, the intention, and the decision-making capacity, as indicated by the definition in Black’s Law Dictionary: “*Suicide is the willful and voluntary act of a person who understands the physical nature of the act, and intends by it to accomplish the result of self-destruction*” [18]. For the purpose of this article, two elements in these definitions deserve scrutiny – the action and the intention.

First, there has to be an action initiated by the patient to cause the own death. Causation is understood in the common legal way, comprising the necessary condition (factual cause) and the sufficient condition (proximate cause) for death to occur [19]. As such, suicide is different from dying through withdrawing and withholding of life-sustaining treatment. If, for example, artificial ventilation is stopped, this may be a necessary condition for dying, but certainly not a sufficient condition, because there has to be a respiratory pathology incompatible with life that exerts its life-terminating effect once ventilation (having temporarily suspended this effect) has been withdrawn.

The suicidal action that causes death does not have to be a positive act; it may also be an omission. It is firmly established in philosophy and law that both acts and omissions can be employed intentionally to cause certain states of affairs and can thus be regarded as forms of human agency [20]. While the most frequent forms of suicide involve positive acts (e.g., gunshot, drug overdose, hanging, and so on), there are also undisputed forms of suicide by omission. Suicidal persons may seek life-threatening situations (e.g., in traffic, water, skydiving) and deliberately refrain from rescuing themselves, even though they could easily do so. If we apply this concept of suicide to VSED, it becomes evident that VSED is a form of suicide by omission [21] – the person’s omission of eating and drinking directly causes death. The cessation of the physiological influx of nutrients and water in VSED parallels the cessation of the physiological influx of oxygen that occurs in hanging or drowning. By contrast, when withdrawing artificial nutrition, hydration, or ventilation, it is not a physiological everyday behavior that is stopped but a medical treatment that technically replaces a pathologically lost organ function.

The second element of suicide that becomes evident in the abovementioned definitions is the intention to kill oneself. This does not mean the intention to allow death to occur naturally (as is the case in withdrawing life-sustaining treatment), but the intention to hasten one’s death. In VSED, the latter intention is clearly present. First, this intention is usually verbalized by the patient towards family members and healthcare professionals [7, 8]. Second, the intention to hasten death is impressively demonstrated by the patient’s resolve to endure hunger and thirst in order to reach this goal. Intention is also the key element that distinguishes VSED as a form of suicide from the alleviation of pain and symptoms with a possible life-shortening effect (sometimes called ‘indirect euthanasia’), which is not a form of suicide. In the latter, patients accept the possibility of a life-shortening effect of high-dosed drugs that are required to treat otherwise uncontrollable symptoms in the dying phase. Irrespective of whether the shortening of life may be a contingent side effect or a necessary means to symptom control, the primary intention is always symptom relief and not death.

VSED should therefore be considered as a form of suicide, as there is both an intention to bring about death and an omission that directly causes this effect. However, there are some characteristics that render VSED a discrete form of suicide, distinct from other forms. Firstly, in contrast to common forms of suicide (e.g., use of firearms, hanging or suffocation, poisoning, falling) [22], VSED is not characterized by an invasive or aggressive act. Second, other methods of suicide typically result in a relatively rapid death, occurring within seconds to minutes, whereas even a complete cessation of eating and drinking will only lead to death after at least several days. As a consequence, the decision to kill oneself can be reversed by resuming eating and drinking [23], at least up to a certain point of no return when the patient loses consciousness or when organ damage is too advanced to save the patient’s life. Moreover, this protracted course and the suffering that may accompany it require more resolve from the patient than quicker forms of suicide, thus providing a better safeguard against impulsive suicidal behavior. Finally, phenomenologically, the dying phase in VSED resembles that of the natural dying process, which also involves some degree of dehydration. This may be a significant advantage for patients as well as relatives and healthcare professionals and may partly explain the widespread acceptance of this practice.

Whilst these three particular characteristics of VSED do not change its status as a form of suicide, they may have an impact on its ethical evaluation. Although suicide is not generally seen as immoral behavior, some features of the various forms of suicide may render them more or less ethically acceptable. For example, jumping in front of a moving train and traumatizing the driver may provoke moral reproach, whereas dying by VSED is not directly harmful to others (although the stress imparted on the relatives by the patient’s protracted death may be significant).

### VSED support and suicide assistance

The question now emerges as to whether the various kinds of support that patients receive in the context of VSED equal to assistance in suicide. In those regions where assisted suicide is, under certain conditions, lawful (e.g., in Switzerland, Oregon, and Washington State), a healthcare professional, family member, or right-to-die association organizes and provides a lethal drug for the patient to swallow or, in the case of swallowing difficulties, an infusion that the patient can deliberately initiate. Two elements of assistance in suicide are critical for our argumentation. First, the assistance is instrumental for death to occur, meaning that, without the assistance, the suicide would not (or could not) occur. Second, the assisting person knows and at least partially shares the patient’s intention to induce death. These two elements are critical when evaluating the medical support that patients can receive in the context of VSED. Four paradigmatic types of scenarios can thus be distinguished:

A. The physician suggests VSED as a way of dying when the patient was unaware of this possibility or did not contemplate it (encouragement).
B. The physician promises to provide symptom relief or any other kind of support after stopping eating and drinking and the patient would choose this way of dying only because of having received this promise beforehand (promise).
C. The patient has already stopped eating and drinking, but would resume oral intake and stop the suicidal process due to suffering if symptom relief or other kinds of support were not provided (support to continue).
D. The patient has stopped eating and drinking and is in need of symptom relief (e.g., because of pain or delirium) or other kinds of medical support but will continue refraining from eating and drinking irrespective of whether this support is provided or not (decision-unrelated support).

**Fig. 1:**
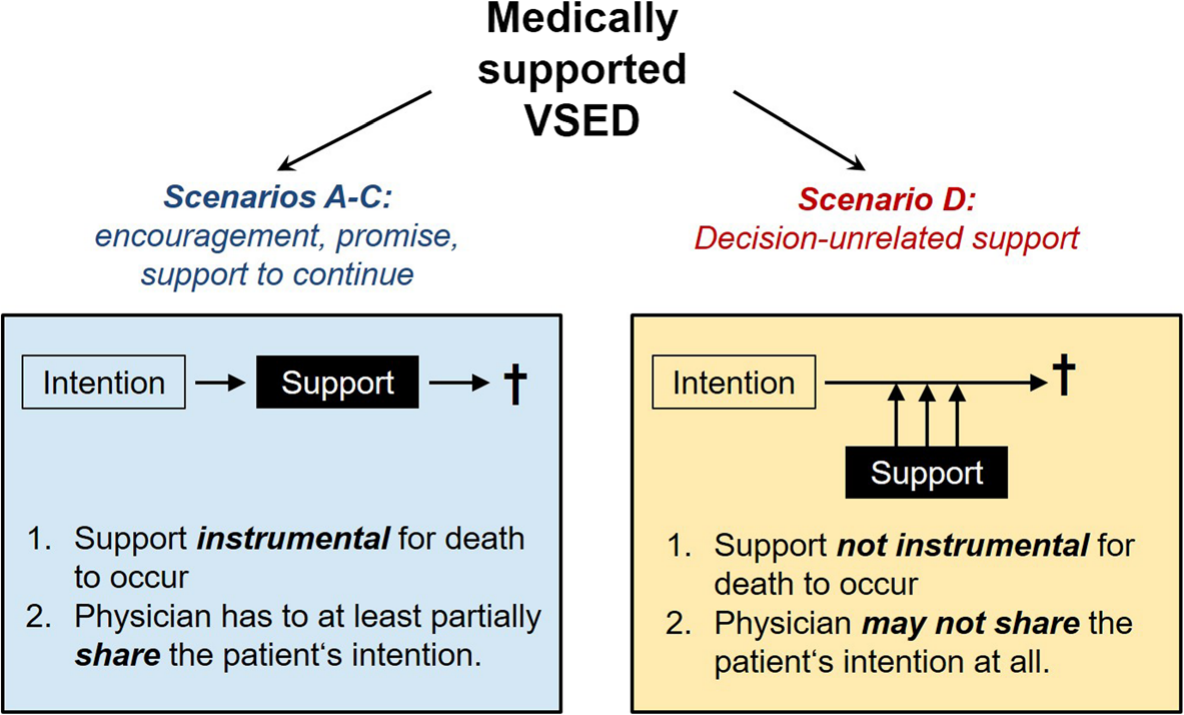
Medically supported voluntary stopping of eating and drinking (VSED): distinction of two ethically divergent types. Scenarios A–C and D are described in the text. † signifies death

Ethically, scenarios A–C differ significantly from scenario D (Fig. 1). In the first three cases, physician support is instrumental to suicide, i.e., support is a necessary condition without which suicide would not occur. It is of secondary importance whether the condition is objectively necessary for suicide to occur, as in scenario A, or subjectively necessary (from a patient’s perspective), as in scenarios B and C. Moreover, in scenarios A–C, physicians know and at least partially share a patient’s intention to hasten death by VSED. In the encouragement case (scenario A), it is evident that physicians would not address the possibility of VSED if they were not prepared to endorse a patient’s intention to choose this option. In scenarios B (promise) and C (support to continue), physicians know that there is a real alternative to providing medical support, namely not embarking on VSED in scenario B or resuming eating and drinking in scenario C. Thus, in scenarios A-C, physicians need to at least partially share a patient’s intention in order to facilitate medically supported VSED. These two elements, instrumental agency and shared intention, imply physician ethical co-responsibility for VSED.

However, in scenario D (decision-unrelated support), physician support is not instrumental to suicide (Fig. 1), because the patient would go ahead anyway. In addition, physicians may well have the sole intention to ease patient suffering and do not have to share the intention to hasten death. Physicians know that, given a patient’s resolve to continue with VSED in any case, there is no real alternative method to ease suffering other than to provide medical support. In contrast to scenario C, this support will not reinforce the decision for VSED and, as such, need not condone a patient’s intention to hasten death.

Thus, there are realistic scenarios (A–C; encouragement, promise, support to continue) in which medical support in the context of VSED fulfils the two critical conditions of suicide assistance, namely sharing the patient’s intention to hasten death and the instrumental nature of the medical act for the initiation or completion of the suicidal act. However, medical support that does not concern VSED but rather the withdrawing or withholding of life-sustaining treatment obviously does not constitute assistance in suicide as treatment limitation is distinct from suicide.

## Conclusions

We have shown that VSED should be regarded as a discrete form of suicide and that medical support in the context of VSED can be equivalent to suicide assistance, depending on the form of support and its relation to the patient’s decision. Our analysis does not presuppose any ethical stance towards the legitimacy of VSED and medical support during VSED – both depend largely on the ethical legitimacy of suicide and suicide assistance, whose discussion is beyond the scope of this article [24, 25]. We do maintain, however, that future ethical discussions on assisted suicide require consideration of medically supported VSED, and vice versa [26].

Thus, the widely held position by palliative care societies, professional bodies of physicians, legal scholars, and ethicists to disapprove of assisted suicide but approve of and even promote medically supported VSED appears inconsistent [11, 12, 25, 27]. With the exception of one situational scenario, both end-of-life decisions should be jointly regarded as being either ethically legitimate or illegitimate. From a legal perspective, those jurisdictions that have legalized assisted suicide under certain procedural requirements may need to apply the same procedural rules to medically supported VSED. Simultaneously, all jurisdictions with laws prohibiting suicide assistance should apply the same laws to medically supported VSED, introduce specific legal regulations pertaining to VSED, or at the very least clarify the legal basis for medically supported VSED. Professional societies in healthcare should strive to harmonize their policies concerning assisted suicide and medically supported VSED. Regardless of their ethical stance, they should all promote a critical, evidence-based and transparent discussion on this clinically and ethically relevant issue.

## Abbreviation

VSED: Voluntary stopping of eating and drinking
